# Shared decision-making and planning end-of-life care for patients with end-stage kidney disease: a protocol for developing and testing a complex intervention

**DOI:** 10.1186/s40814-022-01184-z

**Published:** 2022-10-04

**Authors:** Louise Engelbrecht Buur, Jeanette Finderup, Henning Søndergaard, Michell Kannegaard, Jens Kristian Madsen, Hilary Louise Bekker

**Affiliations:** 1 Department of Renal Medicine, Aarhus University Hospital, Palle Juul-Jensens Boulevard 99, 8200 Aarhus N, Denmark; 2 ResCenPI-Research Centre for Patient Involvement, Aarhus University & the Central Denmark Region, Aarhus, Denmark; 3 Department of Public Health, Aarhus University, Aarhus, Denmark; 4 Department of Clinical Medicine, Aarhus University, Aarhus, Denmark; 5 The Danish Kidney Association, Taastrup, Denmark; 6 Profession School UCN act2learn, Aalborg, Denmark; 7 Leeds Unit of Complex Intervention Development (LUCID), Leeds Institute of Health Science, University of Leeds, Leeds, UK

**Keywords:** End-stage kidney disease, Shared decision-making, Advance care planning, End of life care, Palliative care, Kidney services

## Abstract

**Background:**

Internationally, it has been stressed that advance care planning integrated within kidney services can lead to more patients being involved in decisions for end-of-life care. In Denmark, there is no systematic approach to advance care planning and end-of-life care interventions within kidney services. A shared decision-making intervention for planning end-of-life care may support more effective treatment management between patients with end-stage kidney disease, their relatives and the health professionals. The purpose of this research is to find evidence to design a shared decision-making intervention and test its acceptability to patients with end-stage kidney disease, their relatives, and health professionals in Danish kidney services.

**Methods:**

This research project will be conducted from November 2020 to November 2023 and is structured according to the UK Medical Research Council framework for complex intervention design and evaluation research. The development phase research includes mixed method surveys. First, a systematic literature review synthesising primary empirical evidence of patient-involvement interventions for patients with end-stage kidney disease making end-of-life care decisions will be conducted. Second, interview methods will be carried out with patients with end-stage kidney disease, relatives, and health professionals to identify experiences of involvement in decision-making and decisional needs when planning end-of-life care. Findings will inform the co-design of the shared decision-making intervention using an iterative process with our multiple-stakeholder steering committee. A pilot test across five kidney units assessing if the shared decision-making intervention is acceptable and feasible to patients, relatives, and health professionals providing services to support delivery of care in kidney services.

**Discussion:**

This research will provide evidence informing the content and design of a shared decision-making intervention supporting patient-professional planning of end-of-life care for patients with end-stage kidney disease, and assessing its acceptability and feasibility when integrated within Danish kidney units. This research is the first step to innovating the involvement of patients in end-of-life care planning with kidney professionals.

## Background

The purpose of this research is to outline the research methods we will employ to develop a shared decision-making (SDM) intervention to support patients with end-stage kidney disease (ESKD), relatives, and health professionals (HPs) in planning and deciding about end-of-life care (EoLC) together.

### Integrating EoLC decisions in ESKD: a non-consistent pathway

Core activities of kidney services are to help patients with ESKD preserve residual renal function, initiate kidney replacement therapy when needed, and manage patients’ symptoms. Patients with ESKD may have a high symptom burden, e.g., fatigue, pain, dyspnoea, nausea, decreasing physical function, and restless legs [1]. During progression of their disease, patients make adjustments, or plan changes, to their treatment regardless of whether they are treated with dialysis, conservative kidney management, or a kidney transplant. Kidney care services are central to preparing patients with ESKD for EoLC, and palliative care options, as their health continues to deteriorate. Relatives are often involved in planning care with patients as they fit treatments into their lives; in some cases, patients can lose cognitive function and become unable to make decisions meaning relatives make decisions on their behalf [2, 3]. Patients and their relatives may find these decisions difficult. Being involved in decisions about end-of-life (EoL) options earlier in the illness journey is, on the other hand, associated with an amelioration of worry [4–6].

Internationally, the need for integration of palliative care into nephrology services has been stressed [7–9]. Palliative care aims to enhance patient’s quality of life as they transition from treatment of kidney disease to living well at the EoL [10]. Services differ in the way they offer and integrate palliative care options into patient’s kidney disease management, and some patients die with ESKD before having their palliative care needs fulfilled [11]. Models of how to deliver palliative care within ESKD are limited [12, 13]. Advance care planning (ACP) interventions integrated within kidney services can lead to more patients being involved in decisions about their desired level of treatment, and to an appropriate use of palliative care services [14]. ACP is defined as a process supporting adults at any age or stage of health in understanding and sharing their personal values, life goals, and preferences regarding future medical care [15]. Its goal is to enable patients to receive medical care consistent with their values and preferences during serious and chronic illness [15]. However, there are barriers to implementing ACP within kidney services including HPs’ attitude to talking about EoL issues and fears about taking hope from patients and their relatives [4].

### Preparing a consistent pathway

In Denmark, there is no systematic approach to ACP and integrating EoLC options within kidney services. A systematic review by Brinkman-Stoppelenburg et al. from 2014 [16] found that ACP had a positive impact on the quality of EoLC within different patient populations and settings. The authors concluded that when trying to meet the patient preferences complex ACP interventions could be more effective than written documents alone [16]. However, they stated that existing research on what outcome ACP generates is diverse [16]. An SDM framework may support more effective ACP about kidney disease management and EoLC options being made between patients with ESKD, their relatives, and the HPs. Shared decision-making has been defined as an interpersonal, interdependent process in which patients, their relatives, and HPs relate to and influence each other as they collaborate in making decisions about a patient’s health [17]. In Denmark, SDM interventions [18–20] are an acceptable framework to support patients making dialysis modality decisions earlier in the kidney disease pathway. These SDM interventions are informed from research exploring patient and professional decision-making, and they identify practices that support or hinder patient’s engagement with decisions [21–24]. Shared decision-making interventions are complex interventions with components supporting HPs skills (e.g., decision coaching training), patient understanding (patient decision aids), and patient-professional communications (e.g., consultation prompts).

The aim of this research is to find evidence informing the content of an SDM intervention and to test the acceptability and feasibility of the intervention to patients with ESKD, their relatives, and the HPs in kidney services.

### Study protocol

#### Overall aim of the research

- To develop, and test the acceptability and feasibility of, an SDM intervention to support EoLC planning and decision-making in patients with ESKD, their relatives, and the HPs in kidney services.

#### Primary specific objectives

To identify component parts for inclusion in an SDM intervention supporting EoLC for kidney disease management in Denmark (phase 1), and test its acceptability and feasibility in usual kidney care services (phase 2).

#### Phase 1—research to design the complex intervention

##### Study 1: identifying evidence—literature review

- Scoping review of the literature to identify international primary empirical evidence of evaluated patient involvement interventions for patients with ESKD planning and deciding about their EoLC.

##### Study 2: identifying evidence—patients, their relatives, and HPs’ experiences and needs

- Semi-structured interview study describing multiple-stakeholder experiences of involvement in decision-making and decisional needs towards EoLC.

##### Study 3: developing the prototype SDM intervention

- To iteratively model and develop an SDM intervention using the active elements of the mixed methods investigations from the scoping review and semi-structured interview study, with reference to international guidelines.
- To develop a prototype of the SDM intervention with input from the steering committee.
- To refine the SDM intervention according to the findings and research from study 1 and 2 and the input from the discussions with the experts throughout the developing process to be ready to acceptability testing.

#### Phase 2—acceptability testing of the SDM intervention

##### Study 4: acceptability testing

- To test the acceptability and feasibility of the SDM intervention in patients with ESKD, their relatives, and the HPs making ACP and EoLC decisions within kidney services.

## Methods/design

### Framework informing the research methods

The research design and methods are informed by the UK Medical Research Council (UKMRC) framework for complex interventions development and evaluation. The framework identifies four phases of research: development, feasibility/piloting, evaluation, and implementation [25, 26]. This research project addresses the first two phases: (1) intervention development research, and (2) intervention feasibility/piloting testing, referred to as acceptability testing an evidence-based intervention for use in kidney services. As an application to the above-mentioned framework, the Methods of Researching End of Life Care (MORECare) statement, focusing on EoLC will be used supplementary [8]. This statement is providing 36 best practice solutions for researchers evaluating interventions in EoLC. To assure steady contemplation on implementation of the SDM intervention into kidney services, the phases three and four will be continuously considered throughout the two research phases addressed in this project, as outlined in the MORECare statement [8].

### Framework informing the SDM intervention

The Making Informed Decisions, Individually and Together (MIND-IT) framework by Bekker 2015 [27, 28] provides a representation of the components needed to support different people involved in making healthcare decisions. The International Patient Decision Aid Standards (IPDAS) collaboration checklist [29], the Ottawa Decision Support Framework (ODSF) [30, 31], and the inter-professional shared decision-making model (IP-SDM Model) framework [32, 33] provide the frameworks to carry out this research to identify the constituents of this intervention, and plan for its use and evaluation in practice.

### Steering committee oversight

To get a broad support from relevant stakeholders, an integral part of developing a complex intervention is to create a steering committee [34]. In this research, the purpose of the steering committee is to provide oversight of the research processes and outcomes, to provide rigor and relevance of our outputs to the delivery and experience of kidney care. The steering committee includes the PhD supervisory team, consultants, and nurses from participating nephrology units as well as patients and their relatives with experience of ESKD and EoLC decision-making. Steering committee participants will be recruited through the participating nephrology units by the lead staff, the primary investigator, or the co-investigators. The steering committee will be active in helping include study participants and will be invited to participate in two workshops where the research data will be presented and the steering committee will work on developing a prototype of the SDM intervention.

### Patient and public involvement (PPI) in the research process

Our research team includes a person with ESKD and a relative of a patient with ESKD. Their role is to provide a critical appraisal of the research process from two different perspectives, independent of participants in the research studies. We plan for our PPI members continuously to be involved in the research process and its dissemination through participation as co-authors in publications, in steering committee attendances, as commentators on research materials, and in dissemination activities.

### Settings

The phases 1 and 2 research will be conducted at the Department of Renal Medicine, Aarhus University Hospital (AUH) including satellite units in Randers and Horsens, as well as Herlev and Gentofte Hospitals. From these settings, patients with ESKD, their relatives, and HPs will be involved.

### Recruitment

Patients with ESKD on haemodialysis, peritoneal dialysis, or on conservative kidney management and relatives of patients with ESKD will be considered for participation. Eligible patients will be identified using a clinical provider-initiated indicator, the surprise question (SQ): “would you be surprised if this patient died in the next 12 months?” [35]. The HPs may answer the question on a 5-point Likert scale (“definitely not surprised”, “not surprised”, “neutral”, “surprised” or “very surprised”) [36]. Patients who are cognitively unable to participate will be excluded from the research. Participants will be recruited through the departments and satellite units mentioned above, via relevant staff of the participating nephrology units. A clinical staff member, who knows the patients well, will be asked to approach the patients in person and inform them about the project. If a patient is interested in participating, the primary investigator will contact them and provide them the opportunity to ask any questions they may have related to the research. The lead staff of the participating nephrology units together with the primary investigator and the co-investigators will recruit HPs. Patients, relatives, and HPs will be recruited in the same way for studies in both phases of the research. All participants will be asked to complete written consent prior to enrolment into the research.

### Ethics and dissemination

This project will be conducted in agreement with the ethical principles for medical research involving human subjects [37], and involves informed consent for study participation [38]. We will obtain written consent from patients, relatives, and HPs before we conduct interview studies. No adverse events have been reported from SDM intervention studies [39]. Once we have obtained the planned research data, we will obtain approval from the Danish Data Protection Agency for data management [40]. To support integration of EoLC plans into usual care, dissemination and knowledge transfer will be conducted via peer-reviewed publications, through seminars and conferences, via informed decision-making to patients and their relatives, and by decision coaching training for staff.

### Timeline

The research project will be conducted over a period of three years from November 2020 to November 2023. Month 0–23 will focus on phase 1; month 23–36 will concentrate on Phase 2. From month 12, we will start recruiting patients for the semi-structured interviews and from month 23, patients will be recruited for answering the questionnaires.

#### Phase 1—research to design the complex intervention

In phase 1, the evidence and theoretical frameworks to structure the SDM intervention will be identified though survey designs using literature review and interview methods. This phase will address the service needs and practices for patients with ESKD, their relatives, and the HPs towards making EoLC decisions.

##### Study 1: identifying evidence—literature review

A scoping review following the Joanna Briggs Institute’s (JBI) [41] guidance for conducting scoping reviews will be performed. We will identify published studies evaluating patient involvement interventions for patients with ESKD making EoLC decisions according to the three-step search strategy presented in chapter 11 of the JBI manual for evidence synthesis [42]. An a priori protocol describing the literature search has recently been published [43]. The review will categorize the components included in the patient involvement interventions with reference to their active ingredients, targeted stakeholders, and uncovered potential gaps within the elements of the interventions. Findings will be presented in a data extraction tool developed for the study assisted by a narrative description of how the findings relate to the objective and review questions. Furthermore, it will inform the development of an SDM intervention for Danish kidney services to involve patients with ESKD in EoLC decisions. The Preferred Reporting Items for Systematic Reviews and Meta-Analyses-Scoping Reviews (PRISMA-ScR) statement [44, 45] will guide the review methods to capture data from included studies evaluating patient involvement interventions for patients making EoLC decisions used by patients with ESKD, their relatives, and/or kidney HPs, such as patient decision aids, SDM, ACP education, and difficult conversation packages. The search will be carried out systematically by (LEB) with health information specialists using the search databases; PubMed, Embase, CINAHL, and Scopus. Grey literature will be identified from reference lists of included studies, citations of included studies using ‘Google Scholar’, reviewing the ‘A to Z Inventory’ of patient decision aids included on the Ottawa Hospital Research Institute database [46], and searching public healthcare organization sources by applying a limited number of key words to the ‘Google browser’. Independent of each other, the primary investigator (LEB) and one of the co-investigators (JF) will include and exclude the literature via the systematic review management tool Covidence [47].

##### Study 2: identifying evidence—patients, their relatives, and HPs’ experiences and needs

Individual semi-structured interviews with patients with ESKD, relatives, and HPs in kidney services, will investigate experiences of involvement in the decision-making process, decisional needs, concerns, and values of the participants in relation to planning EoLC when the patients’ health may deteriorate. Sample size of about 12 participants from each stakeholder group, will be recruited using the process described above. The design of the interview guides is based on the interview methods by Kvale and Brinkmann [48] and explore the skills, experiences, reasoning, and motivations of each stakeholder represented within the MIND-IT framework with the Patient Experience of Shared Decision-Making (SHARED) tool [49] will form part of the interview guide. Data from the interviews will be analyzed using a thematic content analysis described by Malterud’s systematic text condensation [50]. This strategy of systematic text condensation to synthesize findings by Malterud is a strategy for qualitative analysis structured upon Amedeo Giorgi’s psychological phenomenological analysis also used towards decision-making [51]. Data will be coded and analyzed in accordance with the four stages of systematic text condensation using the software program NVivo [52]. The evidence gathered from the interview data will be used to inform the design and development of the SDM intervention.

##### Study 3: developing the prototype SDM intervention

The findings will be synthesized with reference to theoretical frameworks associated with supporting patients to make informed, value-based decisions in the context of a worsening disease state, in order to identify the active components within the elements of the reviewed patient involvement interventions and the different stakeholders’ experiences of involvement in decision-making, and decisional needs when planning EoLC. This intervention modelling phase will be supported by methods for developing SDM interventions based on the MIND-IT framework [27, 28], the IPDAS checklist [29], the ODSF [30, 31], and IP-SDM Model [32, 33].

The resulting SDM resources will be presented at the two workshops to be held in September 2022 and October 2022. Here we will work on developing and evaluating a prototype of the intervention for pilot testing. The primary investigator (LEB) will facilitate the two workshops with support from the co-investigators (JF, HB, and JKM). The results from the data analyses of the scoping review and the interview study will form the themes for discussions at the workshops and help elicit the qualified components when developing the prototype of the intervention for pilot testing.

#### Phase 2—acceptability testing of the SDM intervention

In phase 2, the acceptability and feasibility of the SDM intervention will be assessed with patients having ESKD making EoLC decisions, their relatives, and HPs providing the delivery of care in kidney services.

##### Study 4: acceptability testing

A pragmatic, pilot, randomized controlled, non-blinded multicenter superiority trial with two parallel groups will test the acceptability and feasibility of the intervention on patients, relatives, and HPs. The primary endpoint will be decrease in palliative care needs 3 months after receiving the intervention. Secondary endpoints are patient and relative experiences of SDM and HPs conduction of SDM post-intervention. Randomization will be performed as block randomization with 1:1 allocation.

Inclusion criteria will be patients having ESKD who are at a stage in their disease where EoLC has to be considered; age ≥ 18 years old; and alert and oriented. Patients and relatives will be recruited from November 1, 2022 until February 1, 2023. The contact HPs of the eligible patients will be recruiting them and will be allocated to the group where the patients and relatives are allocated. Sample size is dependent on how many patients we are able to recruit to the study by using a clinical indicator and the number of patients willing to participate at the different hospital sites. We hope to recruit around 10 patients for each group.

Bekker’s MIND-IT framework [27, 28] represents the roles of all stakeholders involved in making health decisions and was used to guide the choice of evaluation tools to assess the SDM intervention on patients, relatives, and HPs decision processes and outcomes.

The Integrated Palliative Outcome Score (IPOS)-Renal questionnaire, patient version [53], will be used to measure the primary outcome ‘change in the patient’s palliative care needs. IPOS-Renal is a short measure (11 questions), combining the most common symptoms renal patients experience and additional items from IPOS on concerns beyond symptoms, such as information needs, practical issues, and family anxiety.

The 10-item Patient Experience of Shared Decision-Making (SHARED) tool [49] will measure the degree of which the patients and relatives feel that they have shared in decision-making about their EoLC. Items are rated in a 5-point Likert scale ranging from ‘disagree strongly’ to ‘agree strongly’. Scores are out of 10, with one point for ‘agree’ or ‘agree strongly’, and no points for ‘not sure’, ‘disagree’ or ‘disagree strongly’. Higher scores indicate a more shared decision-making experience.

The Decision Support Analysis Tool (DSAT-10) [54] will be used to evaluate HPs’ use of decision support and thereby their conduction of SDM during the clinical consultation. The DSAT-10 consists of five elements: (1) decision-making status, (2) knowledge of, (3) values/preference associated with others’ involvement in the decision, and (5) next steps. Within these elements, seven assessment criteria are to be fulfilled for the HPs’ decision support to achieve the maximum score of 10 points. The scores out of 10 points are allocated as follows: 1 point, if all checkboxes in a box are checked, except, 2 points, if the importance of both benefits and harms are discussed. Higher scores indicate higher quality of decision support from the HPs. This testing explores if the SDM intervention is acceptable to patients with ESKD, their relatives, and HPs providing services to support delivery of care in kidney and other services (e.g., community-based health care).

Data on gender, age, marital status, and educational level will be collected. The patients’ medical diagnosis and GFR are to be retrieved from their clinical records.

Data will be collected at three time points. Time 1 (T1)—immediately before the commencement of the consultation providing the SDM intervention. Time 2 (T2)—immediately post-intervention, meaning after the SDM intervention in the experimental group, or the usual consultation methods in the control group. Time 3 (T3)—8 weeks after the intervention. Data analysis will be performed using STATA (version 16). Descriptive statistics will be used to summarize the characteristics of the participants: frequency distribution for categorical data, means and standard deviations for continuous data. Characteristics of the participants, such as their demographic profiles, health-related variables, and outcome measures, are compared between groups at baseline (T1). A chi-square test and Fisher’s exact teat are used to examine the difference between the groups in terms of the categorical variables, while Student’s *t* test is used for continuous variables. Intention to treat (ITT) and per protocol (PP) analyses will be conducted. The questionnaire data from both the IPOS-Renal, SHARED, and DSAT-10 will be analyzed according to the guidelines of the individual questionnaires and Cronbach’s alpha methods.

### Intervention development and acceptability testing processes

The intervention development phase will consist of three studies using different methods and the acceptability phase of one study using different methods as outlined in Table 1.

**Table 1:** SDM development and acceptability testing process

Phase/study	Objective	Framework	Method
**1/1**	To synthesise empirical evidence of patient involvement interventions for patients with ESKD making EoLC decisions in Denmark and internationally	UKMRC MORECare JBI PRISMA-ScR	Literature search through the databases; PubMed, Embase, CINAHL, and Scopus and a Grey literature search. Inclusion/exclusion of literature using Covidence. Scoping review of included literature. Synthesising evidence.
**1/2**	To explore experiences and needs for involvement in EoLC for patients with ESKD, their relatives, and HPs	UKMRC MORECare MIND-IT	Present and carry out semi-structured interviews with patients having ESKD, relatives, and HPs. Around 12 patients, 12 relatives, and 12 HPs will be interviewed.
**1/3**	Synthesising the primary and secondary findings from the research and structure a prototype of the SDM intervention	UKMRC MORECare MIND-IT IPDAS ODSF IP-SDM Model	Based on the synthesised evidence through phase 1, study 1 and 2, and supported by methods for developing SDM interventions and relevant theory, a prototype of the SDM intervention will be developed and evaluated during workshops involving expert consensus from the steering committee.
**2/4**	To test if the SDM intervention for planning EoLC is acceptable to patients with ESKD, their relatives and the HPs	UKMRC MORECare MIND-IT IPDAS	A pragmatic, pilot, randomized controlled, non-blinded multicenter superiority trial design will be used to test the SDM intervention in the participating nephrology units. Different questionnaires will be introduced and carried out to evaluate the patient reported outcomes on the SDM intervention, to evaluate the decision support provided by the HPs, and to evaluate if the intervention leads to better healthcare.

*UKMRC* the UK Medical Research Council framework for developing complex interventions, *MORECare* the Methods of Researching End of Life Care statement, *JBI* Joanna Briggs Institute, *PRISMA-ScR* Preferred Reporting Items for Systematic reviews and Meta-Analyses extension for Scoping Reviews Checklist, *MIND-IT* Making Informed Decisions, Individually and Together, *IPDAS* the International Patient Decision Aid Standards guideline, *ODSF* the Ottawa decision support framework, *IP-SDM Model* the interprofessional shared decision-making model

## Discussion

One of the challenges for this research is the lack of prior empirical evidence to inform the structure and content of a complex SDM intervention for EoLC planning and decision-making in patients with ESKD. To the best of our knowledge, this is the first study to develop an intervention for patients with ESKD designed to facilitate EoLC planning and decision-making as part of a SDM process within Denmark and internationally [55, 56]. The findings from the scoping review, the interview studies, and user-centered design workshops with patients, relatives, HPs and experts in patient involvement interventions for kidney disease are essential to ensuring a sustainable intervention is developed for evaluation in kidney services. The research will be carried out in services within Denmark, to ensure the intervention and its implementation is relevant to all stakeholders involved in making this decisions within the Danish kidney care pathway.

One limitation of this research is that it addresses only the first two phases of the complex intervention framework; development and pilot testing [57]. However, evidence to inform the evaluation and implementation phases will be elicited from the proposed research as all studies are grounded within current practice in Denmark, and from multiple stakeholder perspectives.

## Data Availability

Not applicable.

## Abbreviations

SDM: Shared decision-making
ESKD: End-stage kidney disease
HPs: Health professionals
EoLC: End-of-life care
EoL: End-of-life
ACP: Advance care planning
UKMRC: UK Medical Research Council
MORECare: Methods of Researching End of Life Care
MIND-IT: Making Informed Decisions, Individually and Together
IPDAS: The International Patient Decision Aid Standards
ODSF: The Ottawa Decision Support Framework
IP-SDM Model: The Interprofessional Shared Decision-Making Model
PPI: Patient and public involvement
AUH: Aarhus University Hospital
IPOS-Renal: The Integrated Palliative Outcome Scale-Renal
JBI: Joanna Briggs Institute
PRISMA-ScR: The Preferred Reporting Items for Systematic Reviews and Meta-Analyses–Scoping Reviews
SHARED: The Patient Experience of Shared Decision-Making tool
DSAT-10: The Brief Decision Support Analysis Tool
